# Cognition, FDG metabolism, and amyloid accumulation in relation to intracranial arachnoid cysts

**DOI:** 10.1007/s00415-025-13299-3

**Published:** 2025-09-23

**Authors:** Victoria Emilie Neesgaard, Johanne Asperud Thomsen, Jette Stokholm Pedersen, Steen Gregers Hasselbalch, Ian Law, Frantz Rom Poulsen, Tiit Illimar Mathiesen

**Affiliations:** 1 Department of Neurosurgery, Copenhagen University Hospital, Rigshospitalet, Copenhagen, Denmark; 2 Department of Anaesthesiology and Intensive Care, Nordsjællands Hospital, Hillerød, Denmark; 3 Danish Dementia Research Centre, Department of Neurology, Copenhagen University Hospital, Rigshospitalet, Copenhagen, Denmark; 4 Department of Clinical Medicine, Faculty of Health and Medical Sciences, Copenhagen University Hospital, Rigshospitalet, Copenhagen, Denmark; 5 Department of Clinical Physiology and Nuclear Medicine, Copenhagen University Hospital, Rigshospitalet, Copenhagen, Denmark; 6 Department of Neurosurgery, Odense University Hospital, Odense, Denmark; 7 Department of Clinical Research and BRIDGE (Brain Research, Inter Disciplinary Guided Excellence), University of Southern Denmark, Odense, Denmark; 8 Department of Clinical Neuroscience, Karolinska Institutet, Stockholm, Sweden

**Keywords:** Arachnoid cysts, Cognitive symptoms, Brain metabolism, Amyloid accumulation

## Abstract

**Background:**

Though traditionally regarded as harmless, incidental findings, recent literature indicates neuropsychological symptoms associated with intracranial arachnoid cysts (AC). Pathogenesis is unknown, but compression of parenchyma and altered metabolism has been suggested. Patients suspected of dementia often undergo evaluation which can lead to identification of an AC. It is uncertain whether AC can be a primary or contributing cause to symptoms in these and AC have sometimes been raised as a differential diagnosis.

**Methods:**

In this cross-sectional study, patients with AC ≥ 2 cm in a group of 2292 patients referred as part of evaluation for dementia for positon emission tomography (PET) scans with [^18^F]Fluorodeoxyglucose (FDG) and/or Pittsburgh compound B (PiB) were investigated. FDG metabolism, amyloid accumulation, and neuropsychological symptoms were studied when data were available.

**Results:**

The prevalence of intracranial AC ≥ 2 cm was 21 (1%). For 16 (76%) patients, the lesion was supratentorial; for 8 (50%), it was in the left temporal fossa. Neuropsychological symptoms did not correlate with AC localization and did not improve post-surgically. Nineteen (90%) did not have FDG alteration associated with the AC; two (10%) had indication of crossed cerebellar diaschisis. Focal amyloid accumulation around the AC was not found.

**Conclusion:**

In an elderly population of 2292 individuals referred for PET scans under dementia evaluation, 21(1%) had an intracranial AC ≥ 2 cm. Amyloid accumulation and neuropsychological symptoms did not correlate well with cyst localization; a few cases had indication of crossed cerebellar diaschisis on FDG scans. AC are not usually explanatory of cognitive decline in a population investigated for dementia.

## Introduction

With increasing availability and use of CT and MR scanning, incidental findings become more frequent. Arachnoid cysts are traditionally regarded as incidental and harmless and are in most cases left untreated; there are frequent findings with a prevalence of 0.5% as reported in a large meta-analysis on brain scans of people without neurological and psychiatric symptoms, 1.4% as reported in a more recent study of 48,417 patients undergoing MR for various reasons and 2.3% as reported in a study of a large population-based sample of people aged ≥ 70 years [1–3]. They are cystic congenital structures arising from the arachnoid mater and contain cerebrospinal fluid; the most frequent location is the left medial cranial fossa [3, 4]. Despite their status as incidental findings, existing data on arachnoid cysts and symptomatology are contradictory as are recommendations for surgery. Recent literature has described surgically reversible psychological symptoms and cognitive deficits that correlate with cyst locations, also in incidentally detected cysts [4–18]. Moreover, several reports indicate arachnoid cysts as an important differential diagnosis to Alzheimer’s dementia [19–24].

Wester et al. conducted a series of studies on patients referred to neurosurgical departments for evaluation of incidental arachnoid cysts discovered during CT or MR scanning for other indications [5–8, 10, 12–14, 25]. They found psychological and focal cognitive deficits such as attention, memory, spatial abilities, and language that correlated with cyst locations and they found amelioration after surgery. These data were corroborated by several unrelated groups [4, 9, 11, 15–18]. Others suggest that arachnoid cysts contribute to symptoms of other psychiatric or neurological diseases and argue that surgery may ameliorate symptoms despite a persisting underlying condition. In contrast, Rabiei et al. did not find objective cognitive nor psychological deficits to be more prevalent in arachnoid cyst patients nor postsurgical amelioration of symptoms [3, 26]. Likewise, other groups have failed to reproduce findings of symptomatology related to “incidental” arachnoid cysts as well as postsurgical amelioration [27, 28]. It is, thus, controversial whether cysts cause symptoms, whether surgery improves symptoms, and how arachnoid cysts may be mechanistically linked to focal cognitive, psychiatric, or neurological symptoms.

One source of controversy in the debate on arachnoid cysts is the lack of mechanistic understanding of focal cognitive deficits [7, 25]. As arachnoid cysts are considered congenital, they would not be expected to become symptomatic only in adulthood. Moreover, potential agenesis of brain tissue is an unlikely explanation of focal symptoms considering (1) the rapid cognitive normalization that has been reported after surgical treatment, (2) that cognitive symptoms never have shown a statistical association to cyst volume, and (3) that no strict association between symptom alleviation and reduction in cyst volume after surgery has been documented [4, 9, 13, 17, 29].

Recently, interstitial or glymphatic flow has been discussed as a physiological mechanism for nutrient supply and removal of debris [30]. Subsequently, altered pressure gradients could be speculated to affect nutrient supply and removal of debris resulting in metabolic alterations such as accumulation of amyloid. Interestingly, Helland et al. found a significant relationship between intracystic pressure and pre-surgical complaints as well as between intracystic pressure and arterial mean pressure and pCO_2_ [25]. A pressure gradient might obstruct interstitial flow and cause accumulation of metabolites. In addition, Zaatreh et al. found occasional focal metabolic aberrations by FDG-PET scanning [31].

To develop hypotheses that can guide further research, more knowledge on mechanistic findings and different populations with arachnoid cysts is necessary. Case reports and series are likely to reflect bias depending on which patients have been investigated and why. Hence, evaluation of populations that are homogenous with respect to the rationale for scanning such as is the case for the population in the present study can add valuable perspectives.

In the present cross-sectional study, we aimed to determine the prevalence of intracranial arachnoid cysts and to determine if arachnoid cysts were associated with cognitive deficits and changes in brain metabolism.

The specific hypotheses were:

1. The prevalence of intracranial arachnoid cysts in a population of cognitively impaired individuals is higher than the prevalence in the background population.
2. Supratentorial arachnoid cysts can cause focal cognitive deficits which are detectable with a neuropsychological test battery and correlate with cyst location.
3. There are individuals in a population of patients suspected to suffer from dementia that have surgically reversible symptoms caused by supratentorial arachnoid cysts.
4. Supratentorial arachnoid cysts can affect brain metabolism as estimated with FDG-PET imaging.
5. Supratentorial arachnoid cysts can lead to focal amyloid accumulation detectable with PiB-PET imaging.

## Methods and materials

### Design and recruitment

This is a cross-sectional study of intracranial arachnoid cysts in a population of patients undergoing evaluation for dementia. The population includes all adult patients referred from the Memory Clinic at Rigshospitalet, Denmark, between April 9th 2016 and April 9th 2021 for PET scans with FDG and/or PiB as tracers for measuring glucose metabolism and amyloid accumulation, respectively. The standard evaluation at the Memory Clinic includes cognitive screening tests with MMSE, neurological examination, laboratory screening, and a structural scan (CT or MR). Supplementary investigations include neuropsychological evaluation as well as FDG-PET and PiB-PET scanned and clinically evaluated according to recent guidelines [32, 33]. All included patients had available PET scans (all had FDG scans; seven (33%) had PiB scans in addition) and 12 (75%) of the 16 patients with supratentorial arachnoid cysts had available neuropsychological evaluations. Descriptions of CT and/or MR scans were screened for descriptions of intracranial arachnoid cysts and images of cases were reevaluated. Arachnoid cysts were defined as well-circumscribed cysts adjacent to the subarachnoid space displaying cerebrospinal fluid density on CT or MR (i.e., hyperintense on T2 weighted images with FLAIR suppression). Middle cranial fossa cysts were classified according to Galassi [34]. To avoid accidental inclusion of widened subarachnoid spaces, a minimal diameter of 2 cm was set as an inclusion criterion. Available PET scans and neuropsychological data were reevaluated. Patients who had surgery were described in detail.

### Neuropsychological profiles

Two clinical neuropsychologists from the Copenhagen Memory Clinic were presented with the neuropsychological tests scores obtained as part of the dementia evaluation. The patients had been tested with individualized test batteries depending on their age and educational background as well as the specific cognitive complaints leading to referral. The most used neuropsychological tests are listed in Appendix 1.

Apart from the formal test scores, only information about the patients age, gender, and educational level were presented to the neuropsychologists. Information about the patient’s medical history, neurological findings, and other paraclinical investigations which is usually taken into account when doing a clinical neuropsychological evaluation was not provided, and the neuropsychologists were, thus, also blinded to the presence of arachnoid cysts.

The neuropsychologists were asked to rate whether the test scores indicated impairment of specific brain regions: frontal, medial temporal, lateral temporal and parietal areas of the left and right hemispheres as well as the occipital and striatal areas bilaterally. The following scoring system was applied for all ten areas: 0 (no or uncertain impairment), 1 (presumed mild impairment), or 2 (presumed moderate to severe impairment).

**Fig. 1:**
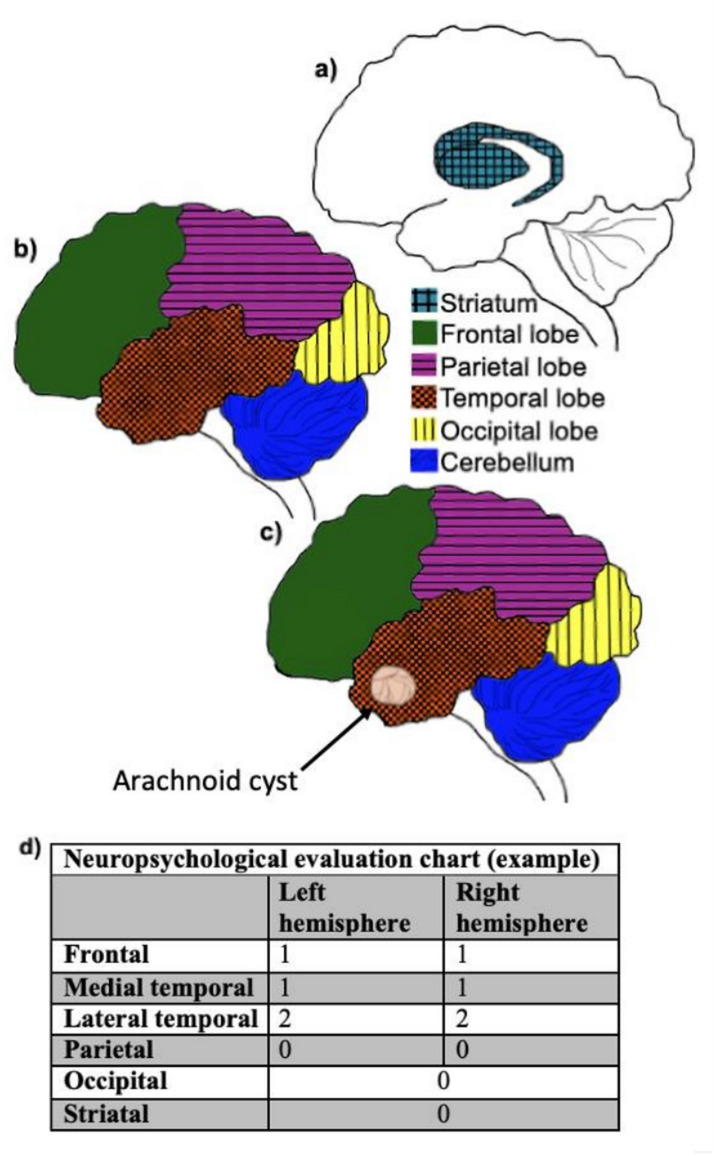
Anatomical areas corresponding to the cognitive cerebral areas and example of a neuro-psychological scoring sheet (graphics created in GIMP). **a** Medial view of the brain. **b** Lateral view of the brain. **c** Example of a cyst in the temporal cognitive cerebral area. **d** The utilized neuropsychological scoring sheet for evaluation of extent of impairment (0–2)

The neuropsychologists initially rated each case individually and then compared these ratings and jointly decided on a score. This rating system was created for the purpose of this study and has not been validated. See Fig. 1.

For temporal arachnoid cysts, the corresponding cerebral area and score were regarded as either the medial temporal area and score or the lateral temporal area and score depending on which score was worst; the area with the most pathological score was chosen because it was presumed that a cyst in the temporal lobe could give rise to focal impairment in either part of the temporal lobe.

### Functional imaging

FDG-PET images were analyzed for differences between signal from the parenchyma surrounding the cyst, contralateral corresponding parenchyma and areas with functional connections to the area of the cyst. PiB-PET images were used to visualize amyloid accumulation in the cortex adjacent to the cyst and comparison to that in the remaining cortex. Analyses were done by an experienced nuclear medicine physician (IL) who was blinded to neuropsychological data.

### Exposure and response variables

Response variables were (1) rating of cognitive dysfunction associated with the cerebral area corresponding to the location of the cyst, (2) specific metabolic alterations as estimated by FDG response around the cyst, and (3) specific accumulation of amyloid around the cyst as estimated by PiB response. The exposure variable was presence of a supratentorial arachnoid cyst.

### Statistics

Results were presented with basic descriptive statistics. Quantitative comparison of cyst prevalence in our population was omitted since qualitative assessment showed extensively overlapping figures.

## Results

### Demographics and inclusion

The study population comprised 2292 patients who had a mean age of 72 years (SD 10 years; range 18;99 years, median 73 years); 1220 (53%) were female.

Of the 2292 patients, 21 (1%) had lesions that fulfilled the imaging criteria for intracranial arachnoid cysts with a diameter of ≥ 2 cm. Of these, 16 (76%) had supratentorial arachnoid cysts and 5 (24%) had infratentorial arachnoid cysts.

**Fig. 2:**
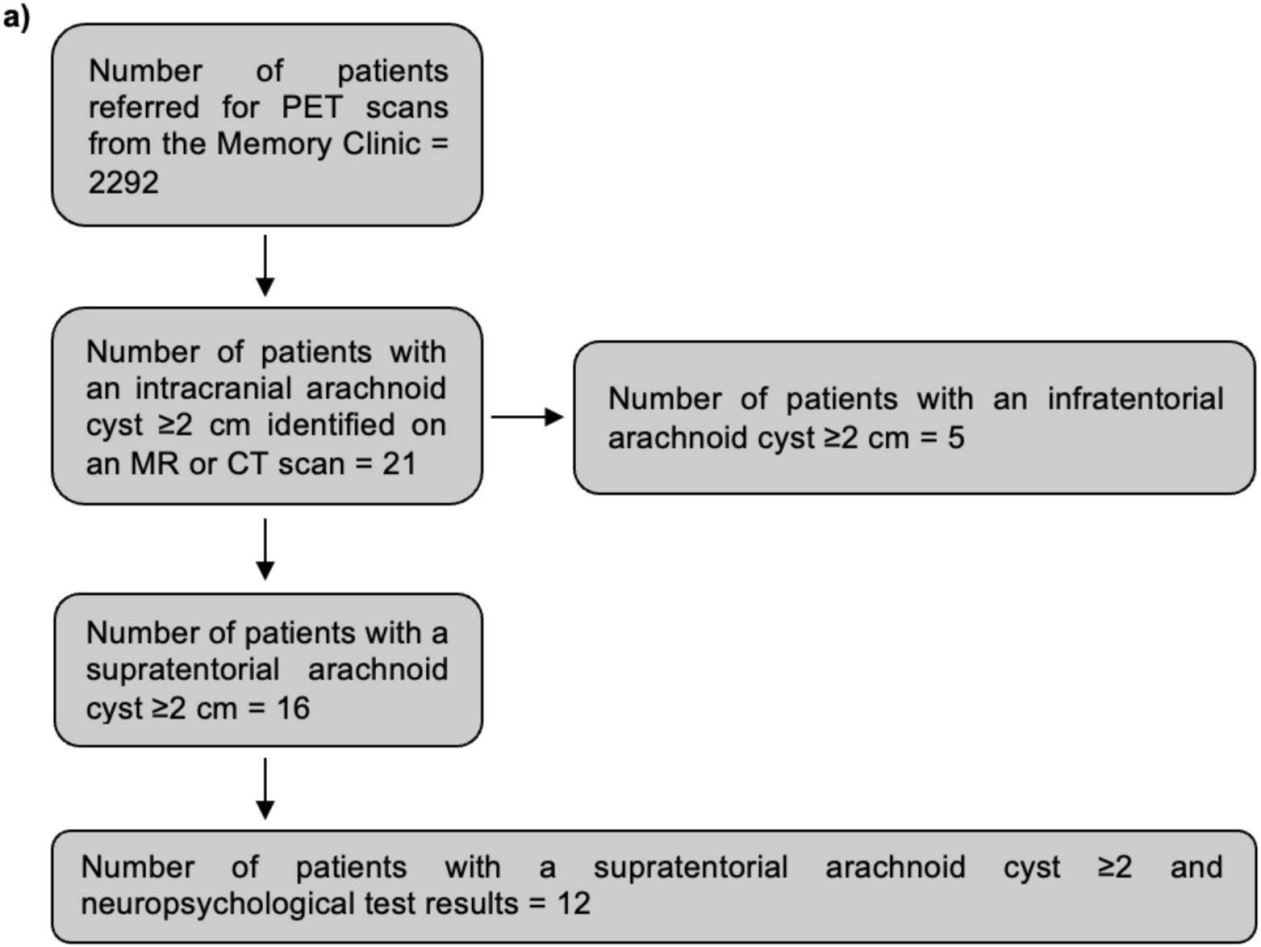
Inclusion

Demographics are shown in Tables 1 and 2. Inclusion is presented in Fig. 2.

**Table 1:** Population characteristics

	Referred population	All identified patients with an intracranial arachnoid cyst ≥ 2 cm	Patients with a supratentorial arachnoid cyst ≥ 2 cm	Patients with a supratentorial arachnoid cyst ≥ 2 cm and available neuropsychological test results	Patients with a temporal arachnoid cyst ≥ 2 cm
Population size: number (%)	2292	21 (1%)	16 (76% of identified arachnoid cysts)	12	11 (52% of identified arachnoid cysts; 69% of supratentorial arachnoid cysts)
Females: number (%)	1220 (53%)	11 (52%)	8 (50%)	7 (58%)	5 (45%)
Age in years: mean (SD^a^), range, median	72 (10), 18;99, 73	72 (8), 53;85, 73	71 (8), 53;85, 73	72 (9), 53;85, 73	71 (9), 53;85, 73
MMSE: mean (SD), range, median	24.3 (4.5), 9;30, 25	25 (3.1), 21;30, 25	25.1 (3.2), 21;30, 25.5	25.8 (3.3), 21;30, 26.5	25.9 (3.3), 21;30, 27

^a^Standard deviation

**Table 2:** Results from individual patients

Patient (sex, age, MMSE)	AC^a^ location	AC volume in cm^3^, Galassi grading	Mass effect	Focal changes in FDG uptake in areas associated with AC localization	Focal changes in PiB response near the AC	Neuropsychological rating of the cerebral area corresponding to AC localization; other impairment	Average neuropsychological rating of the cerebral areas not corresponding to AC localization	Average right hemisphere score	Average left hemisphere score	Final diagnosis
*Supratentorial cysts with available neuropsychological test results, n = 12*										
A (male, 64, 29)	Left temporal lobe	8.5, Galassi type 1	No mass effect	None	N/A^b^	0; impairment of left frontal lobe only (1)	0.1	0	0.25	Sequelae due to intra-cerebral hemorrhage following a head trauma
B (female, 77, 25)	Left frontal lobe	5.1, –	No mass effect	None	N/A	1; bilateral impairment	0.9	1.25	0.5	VD^c^
C (male, 77, 28)	Right temporal lobe	29.0, Galassi type 2	No mass effect	None	N/A	2; bilateral impairment	0.7	1	1	FTD^d^, semantic subtype
D (female, 83, 26)	Left temporal lobe	11.0, Galassi type 1	No mass effect	None	N/A	2; bilateral impairment	1.1	1.5	1.5	LBD^e^, AD^f^ or VD
E (female, 85, 29)	Left temporal lobe	96.0, Galassi type 3	Pre-surgically: compressed sulci, midline shift (5 mm), displacement of left basal ganglia and thalamus Cyst volume increasing from 69 cm^3^ to 96 cm^3^ over 24 months Post-surgically: normalization of parenchyma with disappearance of mass effect	Focal change in right cerebellar hemisphere	N/A	Pre-surgical: 2; bilateral impairment Postsurgical:1; impairment of left hemisphere only	Pre-surgical: 0.1 Postsurgical: 0.2	Pre-surgical: 0.25 Postsurgical: 0	Pre-surgical: 0.5 Postsurgical: 0.75	MCI^g^, probably due to the AC
F (female, 74, 23)	Left temporal lobe	10.4, Galassi type 1	No mass effect	None	N/A	1; bilateral impairment	0.2	0.25	0.5	AD, VD
G (female, 53, 30)	Right temporal lobe	3.2, Galassi type 1	No mass effect	None	N/A	0; impairment of right frontal lobe only (1)	0.1	0.25	0	None given
H (male, 73, 27)	Left temporal lobe	21.6, Galassi type 2	No mass effect	Focal change in right cerebellar hemisphere	N/A	1; bilateral impairment	0.9	1	1	VD
I (male, 73, 28)	Left temporal lobe	4.3, Galassi type 1	No mass effect	None	N/A	0; bilateral impairment	0.6	0.5	0.25	AD or LBD
J (female, 73, 22)	Right parietal lobe	104.4, –	Pre-surgically: compressed sulci, displacement of thalamus Post-surgically: normalization of parenchyma with disappearance of mass effect	None	N/A	2; bilateral impairment	1.1	1	1.25	None given
K (male, 61, 21)	Left frontal lobe	8.3, –	No mass effect	None	N/A	1; bilateral impairment	1	0.75	1.25	None given, probably vascular etiology
L (female, 70, 21)	Left temporal lobe	4, Galassi type 1	No mass effect	None	None	1; bilateral impairment	1	1	1	AD
*Supratentorial cysts without available neuropsychological test results, n = 4*										
M (male, 74, 22)	Right frontal lobe	27.0, –	No mass effect	None	None	–	–	–	–	–
N (male, 67, 23)	Left temporal lobe	20,0, Galassi type 2	No mass effect	None	None	–	–	–	–	–
O (female, 67, 26)	Right frontal lobe	15,5, –	No mass effect	None	None	–	–	–	–	–
P (male, 63, 21)	Right temporal lobe	48.2, Galassi type 2	No mass effect	None	None	–	–	–	–	–
*Infratentorial cysts, n = 5*										
Q (male, 83, 25)	Mid cerebellum	24.4, –	No mass effect	None	N/A	–	–	–	–	–
R (male, 71, 23)	Mid cerebellum	44.1, –	Compression of vermis	None	None	–	–	–	–	–
S (female, 77, 30)	Left cerebellar hemisphere	5.3, –	No mass effect	None	N/A	–	–	–	–	–
T (female, 68, 23)	Left cerebellar hemisphere	8.4, –	No mass effect	None	None	–	–	–	–	–
U (female, 81, 23)	Mid cerebellum	20.1, –	No mass effect	None	N/A	–	–	–	–	-

^a^Arachnoid cyst

^b^Not available

^c^Vascular dementia

^d^Frontotemporal dementia

^e^Lewy body dementia

^f^Alzheimer’s dementia

^g^Mild cognitive impairment

### Neuropsychological data

Neuropsychological test results were available for 12 of the 16 patients with supratentorial arachnoid cysts. Among these were 7 (58%) females; mean age was 72 years (SD 9, range 53;85, median 73) and mean MMSE score was 25.8 (SD 3.3, range 21;30, median 26,5).

All 12 supratentorial arachnoid cyst patients with available neuropsychological data had abnormal neuropsychological scores. Bilateral impairment was found in ten (83%) patients, while unilateral impairment was found in two (17%) patients (patient A and G) and in these, the impaired areas were in the hemisphere containing the cyst. Patient A had a rating of 1 (“mild impairment”) of the left frontal lobe and an arachnoid cyst in the left temporal lobe. Patient G had a rating of 1 (“mild impairment”) of the right frontal lobe and an arachnoid cyst in the right temporal lobe.

### Imaging data

Of the 21 intracranial arachnoid cyst patients, 18 (86%) had no visible signs of compressed tissue, displacement of structures or midline shift, while 3 (14%) (patient E, J and R) had visible displacement of structures.

Of the 21 intracranial arachnoid cyst patients, 19 (90%) did not have FDG response alterations specifically in areas associated with the cyst localization (neither the surrounding cerebral or cerebellar parenchyma nor—for supratentorial arachnoid cysts—the cerebellar parenchyma with functional relation to the cerebral area of the cyst). Existing alterations were found in areas with structural defects and in dementia-like patterns. Two patients (10%) (patient E and H) had reduced response in the cerebellar region with functional relation to the cerebral area of the cyst.

None of the seven intracranial arachnoid cyst patients that had undergone PiB scanning showed specific accumulation of the tracer in parenchyma surrounding the cyst.

### Cases

**Fig. 3:**
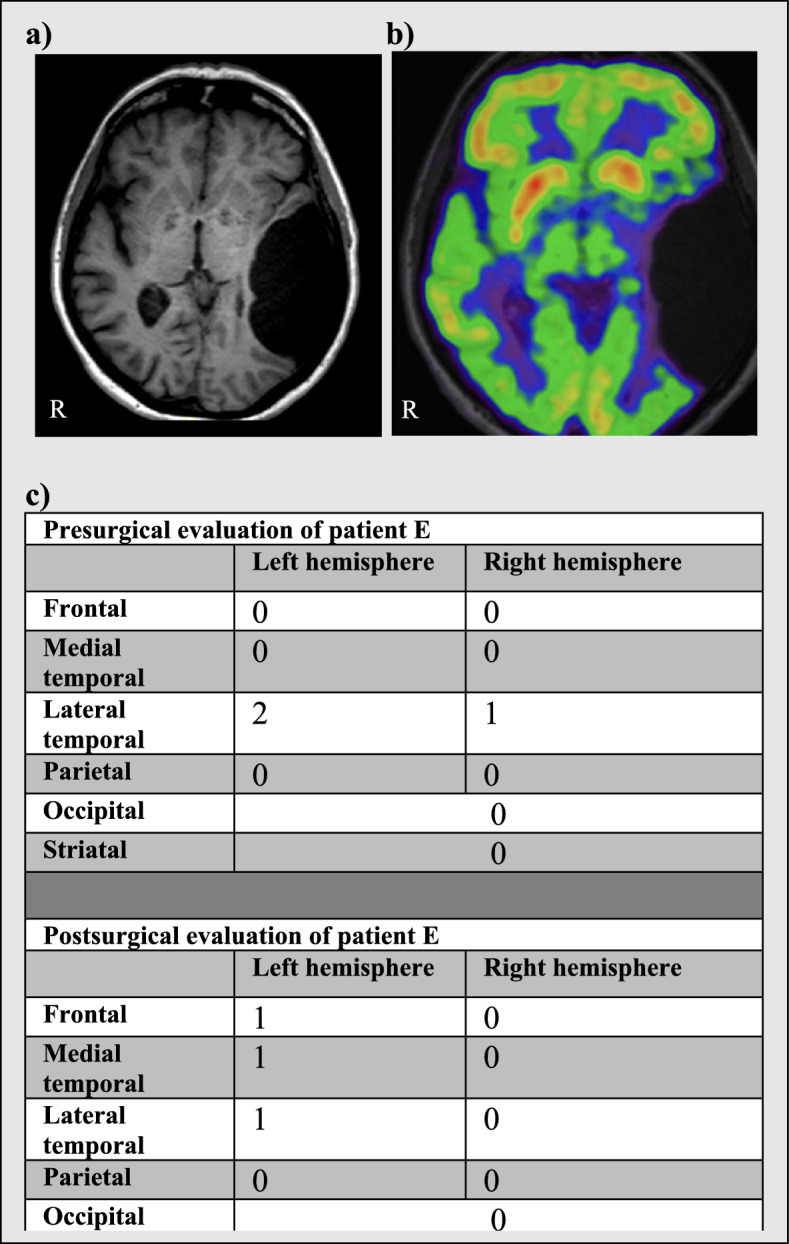
Patient E. Patient E was an 85-year-old female with complaints of impaired memory, word retrieval, reading and writing skills. New MR images revealed that the previously detected arachnoid cyst in the left temporal lobe had expanded from a volume of 69 cm^3^ to a volume of 96 cm.^3^ over the course of 24 months and that a midline shift of 5 mm had developed. It was now classified as a Galassi type 3 arachnoid cyst. An FDG scan showed focally reduced uptake in the right cerebellar hemisphere. Neuropsychological examination indicated impairment of the bilateral temporal areas, most severely in the left hemisphere. The patient had surgery which was followed by disappearance of the mass effect and an extensive reduction of the cyst’s volume on MR. The patient reported subjective improvements of complaints but the neuropsychological evaluation 11 days after surgery did not show clear objective improvement. FDG scanning was not repeated. Dementia was not diagnosed. **a** MR. **b** FDG-PET. **c** Neuropsychological scores (see 2.2 Neuropsychological profiles for explanation)

**Fig. 4:**
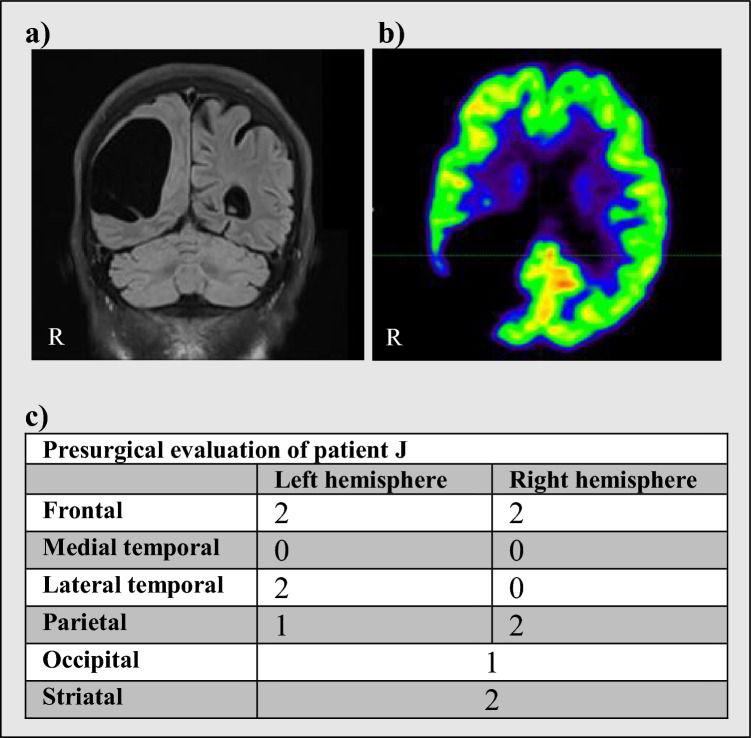
Patient J. Patient J was a 73-year-old female with complaints of memory impairment, problems with word retrieval, troubled spatial orientation, depressive symptoms and anxiety. A right parietal arachnoid cyst had previously been detected and dismissed as asymptomatic. New MR images revealed progression of cyst volume from 61 cm^3^ to 104 cm.^3^ and emergence of a mass effect with compression of surrounding sulci and gyri. The right thalamus was displaced. An FDG scan revealed an increased uptake in the right hemisphere and reduced uptake in the left cerebellar hemisphere. Neuropsychological examination revealed dysfunction of several cognitive domains. The patient had surgery which was followed by normalization of parenchyma on MR but the patient did not experience subjective alleviation of symptoms. Neither neuropsychological examination nor FDG scanning was repeated. The patient was not diagnosed with dementia. a) MR. b) FDG-PET. c) Neuropsychological scores (see 2.2 Neuropsychological profiles for explanation)

Only two intracranial arachnoid cyst patients (patient E and J) were treated surgically. Patient E had neuropsychological reexamination after surgery which did not show any improvement although she reported subjective improvement of non-specific symptoms such as dizziness and brain fogging. Patient J did not have neuropsychological reexamination and did not report any subjective improvement. These patients are presented in Figs. 3 and 4.

## Discussion

The prevalence of patients with intracranial arachnoid cysts with a diameter of ≥ 2 cm was 21 (1%) in this population of 2292 patients referred for PET scans as part of an evaluation for dementia. This was marginally higher than the 0.5% reported in a meta-analysis by Morris et al. considering 15,559 patients without neurological and psychiatric symptoms but marginally lower than the 1.4% reported by Al-Holou et al. and the prevalence of 2.3% reported by Rabiei et al. who studied a population-based sample of people aged ≥ 70 years [1–3]. This finding does not support the hypothesis that the prevalence of intracranial arachnoid cysts in a population of cognitively impaired individuals is higher than the prevalence in the background population. In agreement with previous reports, most (76%) identified intracranial arachnoid cysts were supratentorial and the most frequent location was the left temporal lobe (50%) [3, 4].

Our data did not confirm that supratentorial arachnoid cysts cause focal cognitive deficits detectable with a neuropsychological test battery in a group of patients referred for dementia evaluation. Most patients showed signs of non-lateralized cognitive dysfunction; only two patients (patient A and G) showed lateralized deficits from the hemisphere of the cyst. These deficits were, however, not in clear agreement with cyst location as the presumably impaired cerebral areas were the frontal lobes and the cysts were in the temporal lobes. Only two patients (patient E and J) had their cysts surgically treated but neither had a documented relief of symptoms. This contrasts with the findings in some previous studies but agrees with others as presented above. Still, it should be noted that subtle cognitive deficits caused by arachnoid cysts could be undetectable in this group of patients referred due to suspicion of dementia and that it, therefore, cannot be concluded from this study that a subgroup of arachnoid cyst patients with cognitive symptoms caused by the cysts does not exist nor that symptoms could not be detectable in a healthier population. Studies on different and larger populations would still be warranted to assess a possible causal relation to cognition in patients not affected by dementia.

In the present study, FDG-PET images revealed decreased cerebellar FDG uptake lateralized to the cerebellar hemisphere contralateral to the cyst in two (10%) patients (patient E and H). The decreased cerebellar FDG uptake could be interpreted as reflecting diaschisis resulting from the cyst’s effect on nearby supratentorial brain parenchyma as no other explanation was obvious in these patients. This could indicate that arachnoid cysts can affect brain metabolism; however, further investigation is warranted. The observation generates an interesting hypothesis for prospective studies of diaschisis and arachnoid cysts.

We did not find support for accumulation of amyloid adjacent to the cyst as a sign of impaired interstitial clearance in any of the seven patients with PiB-PET. It is, thus, unlikely that arachnoid cysts in general affect clearance of metabolites such as amyloid from the interstitial compartment.

### Strengths and limitations

A strength of the study is that it reflects a population with a well-defined indication for scanning and well-evaluated cognitive symptoms whereby detection bias was well-controlled. It has high external validity for cognitively impaired, elderly populations. However, the serious comorbidity of the present cohort does leave open the question of the significance of arachnoid cysts in a younger, healthier population as patients under evaluation of dementia already have focal or global cognitive deficits which could mask eventual symptoms related to a cyst. Notably, patients with dementia often have symptoms related to the temporal lobe where arachnoid cysts are found most frequently. Interpretation of cognitive deficits in our population was difficult and subtle cyst-related cognitive effects might be better identified in a healthier population. In addition, the small sample size caused a general lack of power with an associated risk of type 2 errors. We could not apply any statistical tests to evaluate the association between the location of the cysts and the findings on neuropsychological test. The small numbers also affected the assessment of the effect of surgery. Finally, neither patients nor the interviewers performing the tests had been blinded to presence of cysts. The data were obtained during regular evaluation for dementia which may have induced biases. However, knowledge of a potentially symptomatic cyst would primarily be biased towards positive findings, which was contrary to our findings.

## Conclusion

The majority of arachnoid cysts found in a population of cognitively impaired individuals referred for PET scans as part of a dementia evaluation were not symptomatic but seemed to be true incidental findings. FDG metabolism indicated possible affection in the form of crossed cerebellar diaschisis in two patients, while amyloid accumulation adjacent to the cysts was not detected. The largely negative findings suggest that most, but does not prove that all, arachnoid cysts in this population are asymptomatic.

## Data Availability

All available data have been presented in the article. Individual patient related data are protected via GDPR.

## Appendix 1

Most frequently used neuropsychological tests

Rey auditory verbal learning test

Rey complex figure test

Category Cued Recall Test (Danish version, 48 items)

Digit span (forward and backward)

Trail making test A and B

Symbol digit modality test

Stroop color word test

Verbal fluency test

Design fluency test

Boston naming test

Block design

Line orientation test
